# P2X6 Knockout Mice Exhibit Normal Electrolyte Homeostasis

**DOI:** 10.1371/journal.pone.0156803

**Published:** 2016-06-02

**Authors:** Jeroen H. F. de Baaij, Andreas Kompatscher, Daan H. H. M. Viering, Caro Bos, René J. M. Bindels, Joost G. J. Hoenderop

**Affiliations:** Department of Physiology, Radboud Institute for Molecular Life Sciences, Radboud University Medical Center, Nijmegen, The Netherlands; University of the Pacific, UNITED STATES

## Abstract

ATP-mediated signaling is an important regulator of electrolyte transport in the kidney. The purinergic cation channel P2X6 has been previously localized to the distal convoluted tubule (DCT), a nephron segment important for Mg^2+^ and Na^+^ reabsorption, but its role in ion transport remains unknown. In this study, *P2x6* knockout *(P2x6*^*-/-*^) mice were generated to investigate the role of P2X6 in renal electrolyte transport. The *P2x6*^*-/-*^ animals displayed a normal phenotype and did not differ physiologically from wild type mice. Differences in serum concentration and 24-hrs urine excretion of Na^+^, K^+^, Mg^2+^ and Ca^2+^ were not detected between *P2x6*^+/+^, *P2x6*^+/-^ and *P2x6*^-/-^ mice. Quantitative PCR was applied to examine potential compensatory changes in renal expression levels of other *P2x* subunits and electrolyte transporters, including *P2x1-5*, *P2x7*, *Trpm6*, *Ncc*, *Egf*, *Cldn16*, *Scnn1*, *Slc12a3*, *Slc41a1*, *Slc41a3*, *Cnnm2*, *Kcnj10 and Fxyd2*. Additionally, protein levels of P2X2 and P2X4 were assessed in *P2x6*^*+/+*^ and *P2x6*^*-/-*^ mouse kidneys. However, significant changes in expression were not detected. Furthermore, no compensatory changes in gene expression could be demonstrated in heart material isolated from *P2x6*^*-/-*^ mice. Except for a significant (P<0.05) upregulation of *P2x2* in the heart of *P2x6*^*-/-*^ mice compared to the *P2x6*^*+/+*^ mice. Thus, our data suggests that purinergic signaling via P2X6 is not significantly involved in the regulation of renal electrolyte handling under normal physiological conditions.

## Introduction

Adenosine triphosphate (ATP) is an important mediator of cellular communication. In normal physiological conditions, extracellular ATP is involved in a wide variety of cell signaling processes including the recruitment of leukocytes and platelets to damaged cells and sites of increased cell death [1–3]. In the kidney, ATP-mediated purinergic signaling has been linked to renal inflammation [4] and fibrosis [5]. Purinergic signaling is also involved in the regulation of Na^+^ and water transport [6], which means it is one of the factors involved in the onset of hypertension [7–10].

ATP activates both P2X and P2Y receptors [11]. Whereas P2Y receptors are G protein-coupled receptors, P2X receptors form ATP-sensitive ion channels that are expressed at the surface of epithelial cells throughout the body [12–14]. P2X subunits assemble in homo- or heterotrimers with large extracellular loops forming ATP binding sites [12, 15]. Activation of P2X receptors by extracellular ATP results in the non-selective influx of cations into the cell. The P2X family of receptors consists of seven subtypes (P2X1-7) [12, 16]. Within the kidney, P2X receptors are commonly described as inhibitors of ion transport [6]. In mouse collecting duct (CD) cells, ATP release inhibits ENaC mediated Na^+^ uptake [9], which has been linked to P2X2, P2X4, P2X2/6 and P2X4/6 activity. P2 channel activity has also been implicated in the inhibition of water uptake via AQP2 [17].

Recent studies demonstrated that P2X receptors regulate electrolyte reabsorption in the distal convoluted tubule (DCT) [18, 19]. A screening of *P2x* receptors expression levels revealed that P2X4 and P2X6 are the predominant P2X subtypes in the DCT [18]. P2X6 forms heteromers with P2X4 [19, 20]. Recently, P2X4 homomers were found to directly inhibit the apical Mg^2+^ channel transient receptor potential cation channel subfamily M, member 6 (TRPM6). In the DCT, TRPM6 is the main channel for Mg^2+^ reabsorption, and extracellular ATP inhibits Mg^2+^ uptake by increasing P2X-mediated Ca^2+^ influx [21]. However, the role of P2X6 in the regulation of Mg^2+^ transport is still unknown.

The aim of the present study was, therefore, to determine the function of P2X6 in renal electrolyte handling. To this end, *P2x6*^*-/-*^ mice were generated and functionally characterized by measuring serum levels and 24-hour urine excretion of Na^+^, Mg^2+^, K^+^ and Ca^2+^. Potential compensatory mechanisms for loss of *P2x6* gene function were measured by RT-qPCR analysis.

## Methods

### P2X6^-/-^ mice

All experiments were performed in compliance with the Central Animal Laboratory Nijmegen and the animal ethics board of the Radboud University Nijmegen (DEC #2013–185). *P2x6*^*+/-*^ mice (mus musculus), from a mixed background, (B6J-129P2-P2RX6) were provided by The Mary Lyon Centre, Oxfordshire, UK. The acquired *P2x6*^*-/-*^ mice were backcrossed once with C57BL/6 wild-type. 6–10 littermates were housed in standard cages with bedding material consisting of straw and paper in a temperature- and light-controlled room with standard pellet chow and deionized drinking water available *ad libitum* (S1 ARRIVE Checklist). To confirm full loss of P2X6, PCR amplification of the complete *P2x6* transcript was performed in isolated mouse heart cDNA. PCR products were designed to be exon-exon junction spanning and amplify several exons in a range of 145–564 bp in ascending order. PCRs were performed with Amplitaq Gold 360 mastermix (Invitrogen, Bleiswijk, The Netherlands) in a Biometra T3000 (Westburg, Leusden, The Netherlands). Primer sequences are described in S1 Table.

### Phenotyping

Phenotyping was performed on *P2x6*^+/+^, *P2x6*^+/-^ and *P2x6*^-/-^ mice to assess differences in behavior and phenotype. Body condition scoring (BCS) was performed as described previously [22]. Mice were checked for abnormal breathing, grooming and fur condition. Additionally, mice were closely monitored for the ability to support their own body weight and whether they lacked voluntary movement of the fore and hind limbs. Eyes and ears were visually inspected for abnormalities. All mice were thoroughly investigated for fighting wounds. Furthermore, animals were checked for bearing a possible rectal or vaginal prolapse. Activity and curiosity of the animals was verified by assessing open-field behavior as described previously [22].

### Experimental set-up

The experimental sample size was determined by a power calculation (N≥2σ(Zα+Zβ)^2^/δ2), based on an estimated urinary Mg^2+^ excretion of 65 ± 35 μmol/day [23]. A difference of 70% (45 μmol/day) (δ) with a standard deviation of 75% (48 μmol/day) (σ) in urinary Mg^2+^ excretion was expected, combined with a power of 80% (β) and a significance threshold of 5% (α) this results in a minimum sample size of 10 animals (N) per group. For the experiment, 30 adult male mice (10 *P2x6*^*-/-*^ (knock-out), 10 *P2x6*^*+/-*^ (heterozygous) and 10 *P2x6*^*+/+*^ (wild-type)) were housed individually in mouse metabolic cages during the last 48 hrs for urine and feces collection (24 hrs adaptation, 24 hrs sampling). Littermates were allocated to an experimental group based on genotype. Animals were 8–10 weeks old. Blood samples were collected by orbita extraction in isoflurane-anesthetized mice during the day. The primary outcome measure was changes in urinary Mg^2+^ excretion levels between experimental groups. Secondary measures were differences in other electrolytes, such as Na^+^, K^+^ and Ca^2+^. After being anesthetized with 4% (v/v) isoflurane, all thirty mice were sacrificed. Blood samples, kidneys and hearts were collected and the organs were immediately frozen in liquid nitrogen.

### Expression profiling

Total RNA was isolated using TRIzol total RNA isolation agent (Invitrogen, Bleiswijk, the Netherlands) according to the manufacturer’s protocol. Obtained RNA was treated with DNase (Promega, Fitchburg, WI, USA) to remove genomic DNA. Subsequently, reverse transcription of the mRNA by M-MLV reverse transcriptase (Invitrogen, Bleiswijk, the Netherlands) was performed for 1 hr at 37°C. Gene expression levels were determined by RT-qPCR on a BioRad (Hercules, CA, USA) analyzer using SYBR Green and normalized for *glyceraldehyde 3-phosphate dehydrogenase* (*Gapdh)* expression levels. Primer sequences are provided in S2 Table.

### Electrolyte measurements

Serum and urinary total Mg^2+^ and Ca^2+^ concentrations were determined using a xylidyl blue colorimetric assay kit according to the manufacturer’s protocol (Roche/Hitachi, Tokyo, Japan). In short, serum Mg^2+^ and Ca^2+^ were measured photometrically via the decrease in xylidyl blue absorbance. 1 M Mg^2+^ and Ca^2+^ standards (Sigma Aldrich, Zwijndrecht, The Netherlands) were used to generate standard dilution curves. The assay was calibrated using a Mg^2+^ Precinorm (Sigma Aldrich, Zwijndrecht, The Netherlands) with a concentration of 0.76 mM Mg^2+^. Samples were measured on a Nanodrop 2000c spectrophotometer (Thermo Scientific, Breda, The Netherlands) at 600 nm. 20 μl of urine was acidified by adding 4 μl 1M HCl and diluted 30x with MQ before performing the Mg^2+^ and Ca^2+^ assays. All feces were dissolved in 5 ml 65% v/v sulfuric acid and incubated for 10 min. at 50°C and then diluted 50 times in MQ before use. Serum and urinary Na^+^ and K^+^ concentration were determined by the Radboudumc clinical lab on an automated system according to the manufacturer's protocol (Abbott Diagnostics, Belgium).

### Isolation of membrane and cytosol fractions

Membrane and cytosol fractions were isolated from kidney tissue of P2x6^-/-^ and P2x6^+/+^ littermates by ultracentrifugation. To this end, half of a mouse kidney was first homogenized using a homogenizer (bedrijf) in 1 ml of lysis buffer (50 mM Tris pH 7.5, 1 mM EDTA, 1 mM EGTA, 1 mM NaOrthovandate, 5 mM NaF, 5 mM glycerolphosphate 0.27 M sucrose) containing 1 tablet of complete protease inhibitor cocktail (Roche) and 0.1% b-mercapto-ethanol without detergents. Samples were than centrifugated at 3000 g for 10 minutes at 4°C. The supernatant was then centrifugated at 100,000 g for 1 hour at 4°C in a Sorvall™ WX Floor Ultra Centrifuge (Thermo Scientific, Asheville, NC, USA) with a 70.1Ti rotor. The supernatant was taken and proteins in the cytosolic fraction were solubilized by adding 1% NP-40. The remaining pellet was resuspended in 1 ml lysis buffer without b-mercapto-ethanol and centrifuged at 100,000 g for 15 minutes at 4°C to get rid of all cytosolic parts. The remaining pellet consists of the membrane fraction and is subsequently resuspended in 100 μl lysis buffer containing 1% NP-40 and b-mercapto-ethanol. To remove any debris, membrane fractions were spun a final time at 12,000 g for 10 minutes at 4°C. The remaining supernatant was used for further experiments.

### Cell culture and transfection

HEK293 cells were grown in Dulbecco’s modified Eagle’s medium (Lonza, Leusden, The Netherlands) containing 10% (v/v) fetal calf serum (FCS), 2 mM L-glutamine at 37°C in a humidity controlled incubator with 5% (v/v) CO_2_. The cells were transiently transfected with the respective construct using Lipofectamine 2000 (Invitrogen Bleiswijk, The Netherlands) in a 1 μg DNA to 2 μl lipofectamine ratio and analyzed on Western blot, 48 hours after transfection. Cell samples were lysed in lysis buffer (50 mM Tris pH 7.5, 1 mM EDTA, 1 mM EGTA, 1 mM NaOrthovandate, 5 mM NaF, 5 mM glycerolphosphate 0.27 M sucrose and 1 tablet of complete protease inhibitor cocktail (Roche)) containing 1% (v/v) Triton X-100.

### BCA protein measurements and western blotting

Protein concentrations were measured with a colorimetric Pierce BCA protein assay kit (ThermoFisher scientific, Breda, The Netherlands) according to the manufacturer’s protocol. In short, samples were loaded on a 96 well plate, incubated for 30 minutes at 37°C and measured at 562 nm with a Biorad Benchmark plus microplate photospectrometer (Biorad, Veenendaal, The Netherlands). Protein lysates of membrane and cytsolic fractions of P2x6^-/-^ and P2x6^+/+^ mouse kidney samples were denatured in Laemmli containing 100 mM DTT for 30 minutes at 37°C. Samples were then subjected to SDS-PAGE and Western blots were incubated with a rabbit anti-mouse P2x2 antibody (Alomone Labs, Jerusalem, Israel, 1:500) or a rabbit anti-mouse P2x4 (H-40, Santa Cruz biotechnology, Santa Cruz CA, USA, 1:500) in 5% milk o/n at 4°C. Then, immunoblots were incubated 45 minutes at RT with peroxidase conjugated goat anti-rabbit secondary antibodies (Sigma-Aldrich, Zwijndrecht, The Netherlands, 1:10,000). β-actin was used as a loading control and samples were stained in 5% milk o/n at 4°C with anti-mouse β-actin (Sigma-Aldrich, Zwijndrecht, The Netherlands, 1:10,000). HEK293 cells, which were transiently transfected with HA-tagged pCINEO-hP2X4-IRES-GFP and pCINEO-mock-IRES-GFP constructs, were subjected to the same procedure as described above.

### Statistical analysis

All results are depicted as mean *±* standard error of the mean (SEM). The statistical analyses were conducted by one-way ANOVA, followed by a Tukey’s post hoc test when comparing the three treatment groups (n = 10 for each group). Difference in means with *P* values < 0.05 were considered statistically significant.

## Results

### Breeding of *P2x6*^*-/-*^ mice

**Fig 1:**
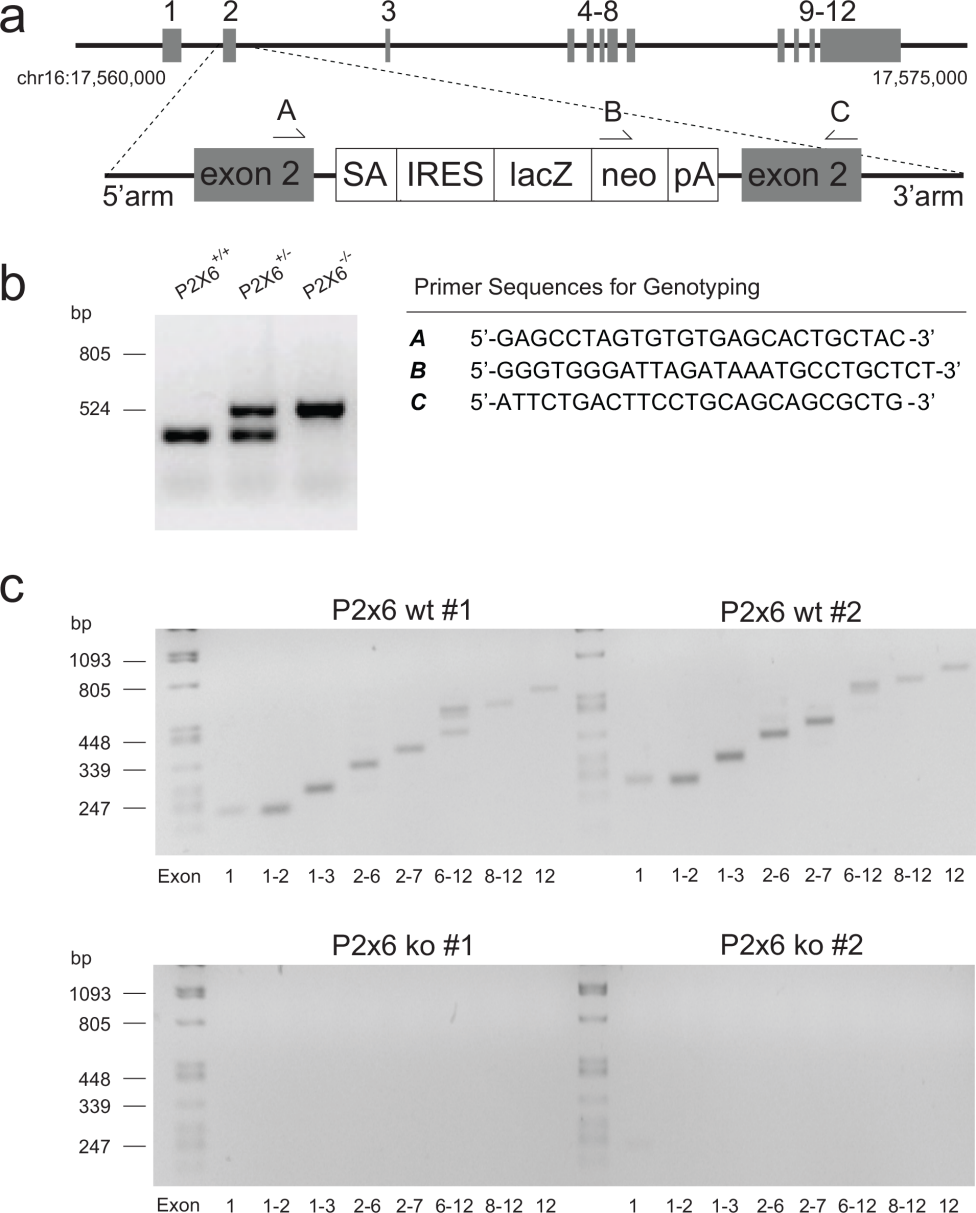
Characteristics of the *P2x6*^*-/-*^ mouse. a) Targeted insertion of the knockout (KO) cassette. Top: *P2x6* locus on chromosome 16. Bottom: targeted allele in which the KO cassette is inserted within exon 2. Grey boxes indicate exons, arrows depict genotype primers. a-c) SA: Splice acceptor site, IRES: internal ribosome entry site, LacZ: ß-galactosidase, NEO: neomycin cassette, pA: polyA. b) Identification of the mouse genotype by PCR analysis of ear-derived DNA. The PCR product size ± 478 bp shows the presence of the wild-type allele (+/+), using primers A and C; the PCR product sized ± 800 bp shows the KO allele (-/-) using primers B and C. Both alleles are detected in heterozygous animals (+/-). c) cDNA isolated from murine heart samples were used to amplify exons 1–12 of *P2x6* with PCR. The top agarose gels show the PCR products for exons 1–12 in two *P2x6*^*+/+*^ animals. The lower gels represent the PCR products for exons 1–12 in two *P2x6*^*-/-*^ animals.

To assess the function of P2X6 *in vivo*, *P2x6*^-/-^ mice were generated (Fig 1A). Inactivation of the *P2x6* gene was achieved by inserting a LacZ knockout (KO) cassette in exon 2 of the gene. The breeding of *P2x6*^*+/-*^ mice resulted in a normal Mendelian inheritance pattern in offspring. Of a total of 116 mice, 23% were genotyped *P2x6*^*+/+*^, 49% *P2x6*^*+/-*^ and 28% *P2x6*^*-/-*^. All offspring were genotyped for the insertion of the knockout cassette and presence of the wild type *P2x6* allele (Fig 1B). Mouse heart cDNA was used to establish the presence of full-length *P2x6* transcripts in *P2x6*^*+/+*^ mice and the complete absence of *P2x6* expression or potential alternatively spliced transcripts in *P2x6*^*-/-*^ animals (Fig 1C). The expected PCR products were present in *P2x6*^*+/+*^ mice and not detectable in the *P2x6*^*-/-*^ animals.

### Normal behavior in *P2x6*^*-/-*^ mice

*P2x6*^+/+^, *P2x6*^+/-^ and *P2x6*^-/-^ mice were subjected to an inspection of behavior and phenotype according to the guidelines for assessing the health and condition of mice [22]. All genotypes had a BCS of 3, indicating that all animals were in optimal condition. No changes in breathing, grooming and fur quality were observed. Mice exhibited no problems with any voluntary movement or with supporting their own body weight. Both eyes and ears were normal in all genotypes. All mice displayed normal open field behavior. No fighting wounds could be detected after thorough examination and there were no mice with a rectal or vaginal prolapse.

### Normal renal electrolyte handling in *P2x6*^*-/-*^ mice

**Fig 2:**
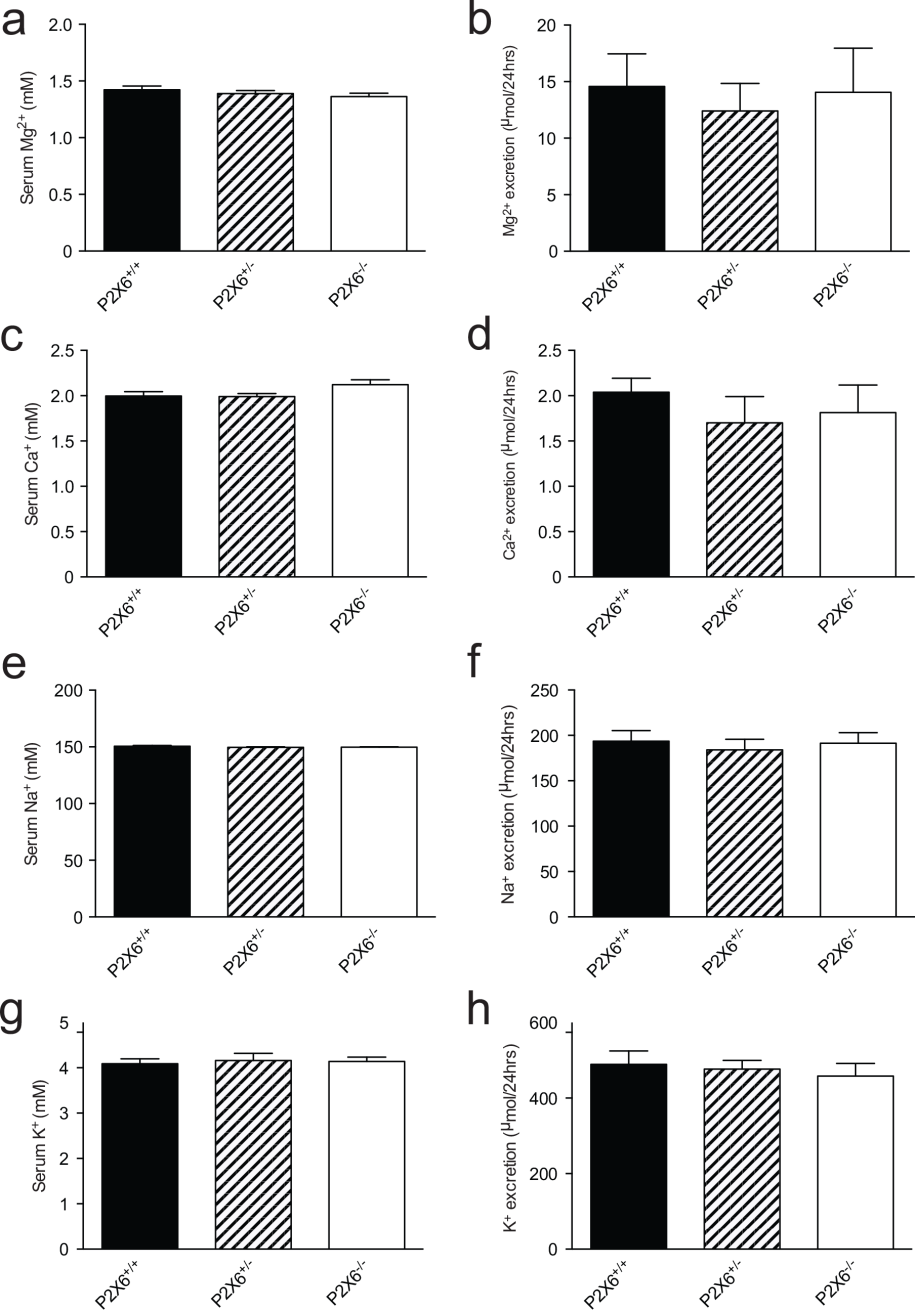
Normal renal electrolyte handling in *P2x6*^*-/-*^ mice. a) Serum Mg^2+^ concentrations of wild-type, heterozygous and knockout P2x6 mice. b) 24 hrs urinary Mg^2+^ excretion of wild-type, heterozygous and knockout P2x6 mice. c) Serum Ca^2+^ concentrations. d) 24 hrs urinary Ca^2+^ excretion. e) Serum Na^+^ concentrations. f) 24 hrs urinary Na^+^ excretion. g) Serum K^+^ concentrations. h) 24 hrs urinary K^+^ excretion. Values (n = 10) are presented as means ± SEM.

To investigate the role of P2X6 in renal electrolyte handling, blood, 24 hrs urine and feces were collected using metabolic cages. No significant differences in body weight, food and water intake were identified between *P2x6*^*+/+*^, *P2x6*^*+/-*^ and *P2x6*^*-/-*^ mice littermates (Table 1). Furthermore, urine production did not significantly change between the three mice groups. A similar trend was observed in the feces production, which was not significantly altered. Because P2X6 is localized to the DCT, changes in electrolyte handling were expected for Na^+^ and Mg^2+^. However, measurements of serum (Fig 2A and 2E) and 24 hrs urinary excretion (Fig 2B and 2F) showed that all groups have similar Na^+^ or Mg^2+^ concentrations and excretion rates, respectively. Furthermore, serum and 24 hrs urine excretion of Ca^2+^ and K^+^ remained unchanged regardless of genotype (Fig 2C, 2D, 2G and 2H).

**Table 1:** Metabolic Parameters of P2x6 Mice.

	*P2x6*^*+/+*^	*P2x6*^*+/-*^	*P2x6*^*-/-*^
**Body weight (g)**	21.5 ± 0.9	21.9 ± 1.0	21.6 ± 1.0
**Water intake (mL)**	4.1 ± 0.1	4.8 ± 0.2	4.7 ± 0.2
**Food intake (g)**	3.4 ± 0.3	3.9 ± 0.1	3.9 ± 0.1
**Urine volume (mL)**	1.2 ± 0.1	1.2 ± 0.1	1.3 ± 0.1
**Feces weight (g)**	1.4 ± 0.1	1.6 ± 0.0	1.7 ± 0.1

Body weight, water intake, food intake, urine volume and feces weight was assessed after housing the animals for 48 hrs in metabolic cages. Numbers represent the mean ± SEM.

### Gene expression of renal electrolyte transporters is not altered in *P2x6*^*-/-*^ mice

**Fig 3:**
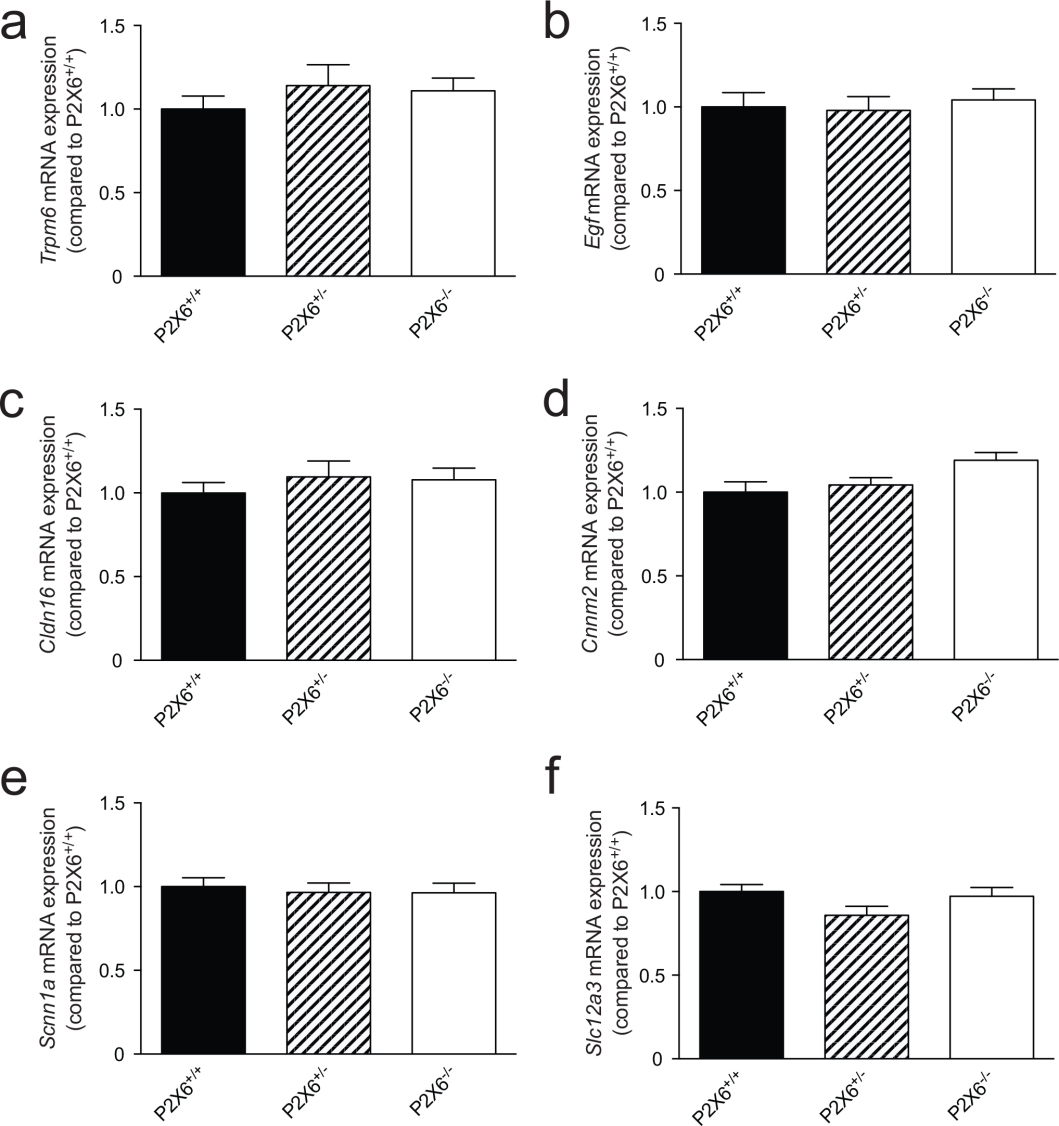
Gene expression of renal electrolyte transporters was not altered in *P2x6*^*-/-*^ mice. a-f) The mRNA expression levels of *Trpm6* (a), *Egf* (b), *Cldn16* (c), *Cnnm2* (d), *Scnn1a* (e), *Slc12a3* (f) in kidney of *P2x6*^*+/+*^ (Black bars), *P2x6*^*+/-*^ (Striped bars), *P2x6*^*-/-*^ (white bars) mice were measured by quantitative RT-PCR and normalized for *Gapdh* expression. Data (n = 10) represent mean ± SEM and are expressed as the fold difference when compared to the expression in *P2x6*^*+/+*^ mice.

**Fig 4:**
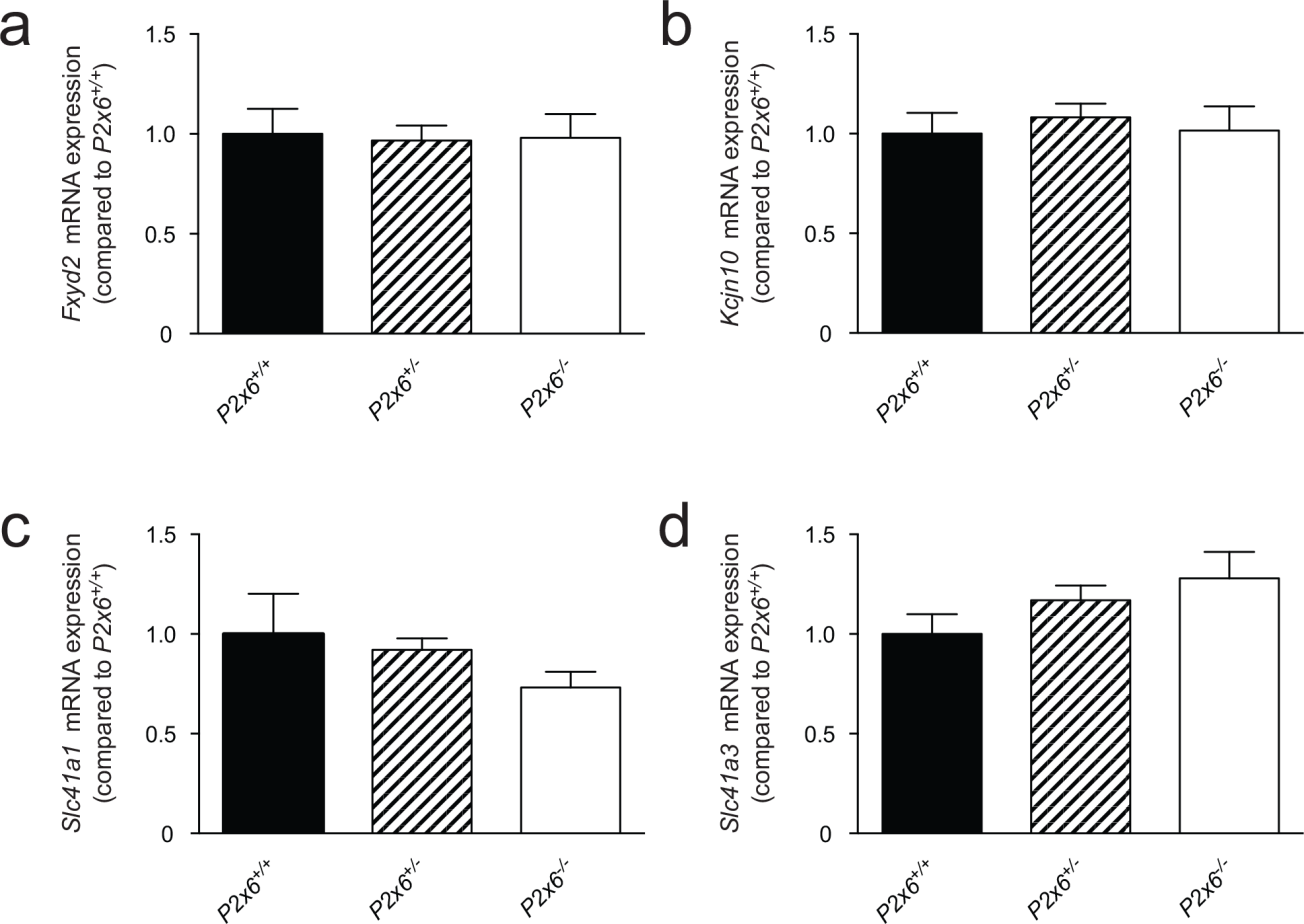
*P2x* subunit expression in response to the loss of *P2x6* function in the kidney. a-f) The mRNA expression levels of *P2x1* (a), *P2x2* (b), *P2x3* (c), *P2x4* (d), *P2x5* (e), *P2x7* (f), in kidney of *P2x6*^*+/+*^ (Black bars), *P2x6*^*+/-*^ (Striped bars), *P2x6*^*-/-*^ (white bars) mice were measured by quantitative RT-qPCR and normalized for *Gapdh* expression. Data (n = 10) represent mean ± SEM and are expressed as the fold difference when compared to the expression in *P2x6*^*+/+*^ mice.

**Fig 5:**
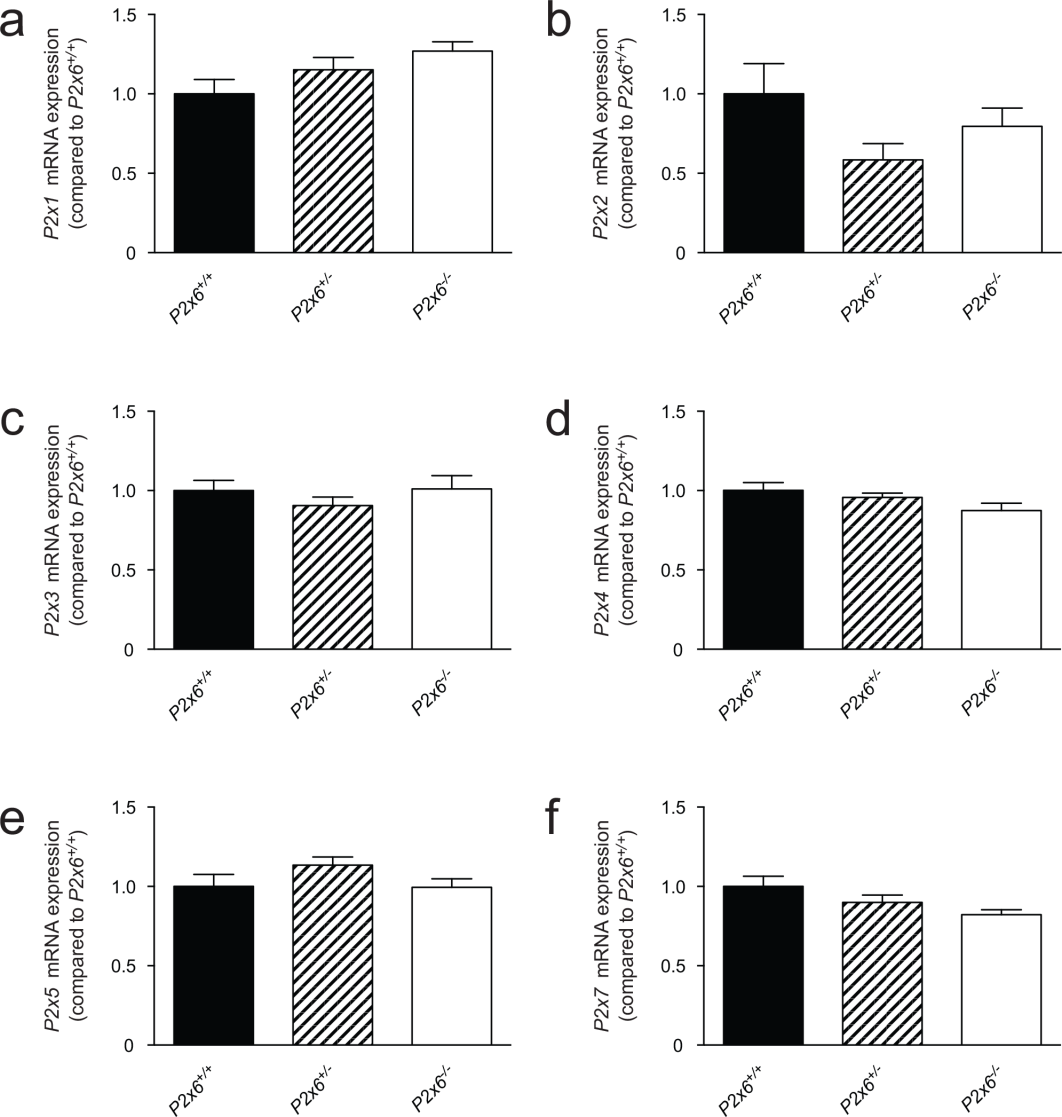
Basolaterally expressed compensatory mechanisms for the loss of P2x6 function in the kidney. a-d) The mRNA expression levels of *Fxyd2* (a), *Kcjn10* (b), *Slc41a1* (c), *Slc41a3* (d) in kidney of *P2x6*^*+/+*^ (Black bars), *P2x6*^*+/-*^ (Striped bars), *P2x6*^*-/-*^ (white bars) mice were measured by quantitative RT-qPCR and normalized for *Gapdh* expression. Data (n = 10) represent mean ± SEM and are expressed as the fold difference when compared to the expression in *P2x6*^*+/+*^ mice.

**Fig 6:**
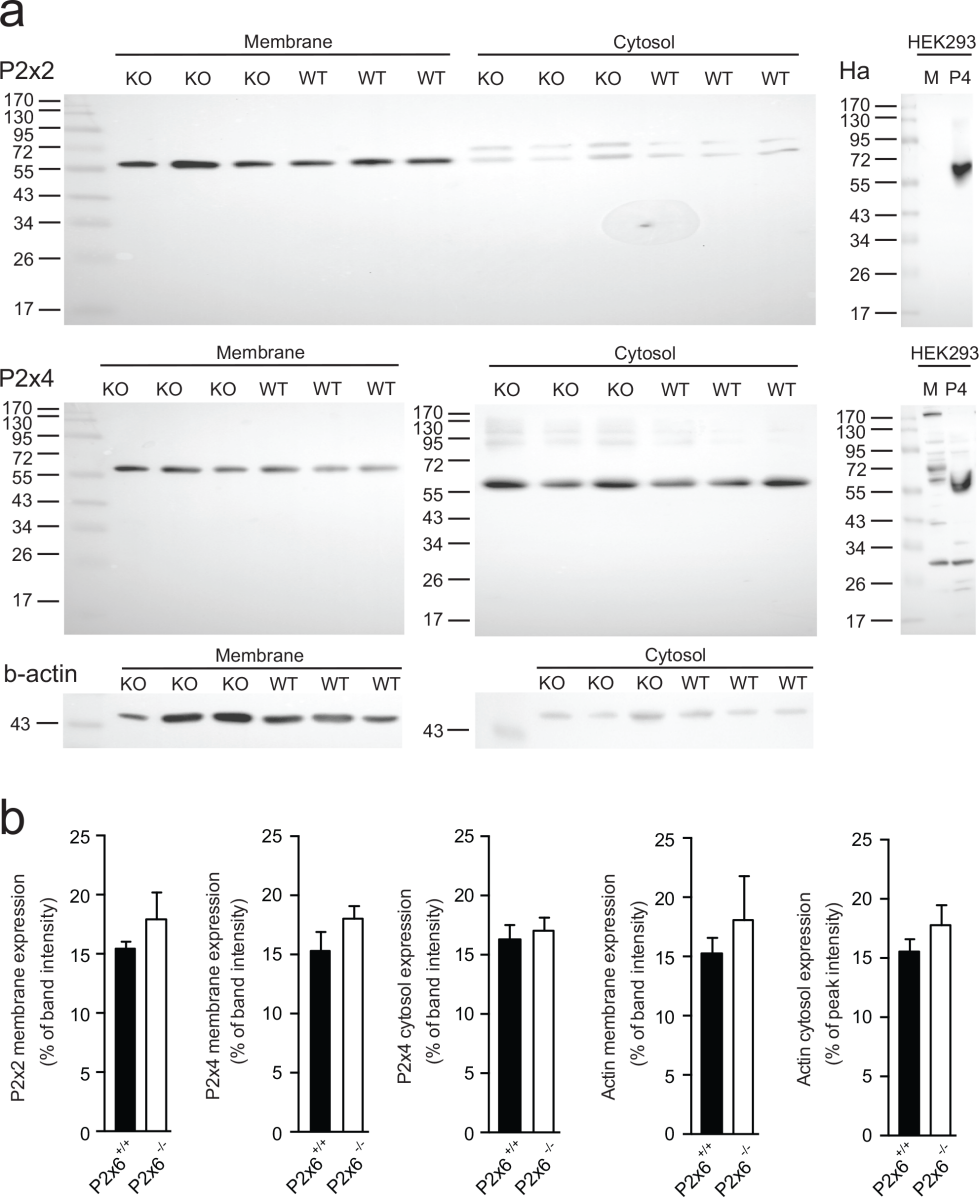
Protein abundance of P2x2 and P2x4 in response to the loss of P2x6. a) Western blots of membrane and cytosol fractions of *P2x6*^*-/-*^ (KO) and *P2x6*^*+/+*^ (WT) mice. The upper blot shows the immune-staining for P2x2 in mouse kidney material. To the right a western blot of HEK293 cells transiently transfected with HA-tagged P2x4 (P4) and mock (M) constructs is displayed. Middle, two western blots below are immune-stained for P2x4, left depicts *P2x6*^*-/-*^ (KO) and *P2x6*^*+/+*^ (WT) material stained for P2x4, right represents a P2x4 blot on HEK293 material transiently transfected with human P2X4 and a mock construct. Bottom, displays a ß-actin immune-staining used as a loading control. Ladders (ez-run prestained marker (ThermoScientific, Breda, The Netherlands) represent protein size in kilo Dalton (kD). b) Protein expression levels for the P2x2 membrane lysate, P2x4 membrane lysate, P2x4 cytosol lysate, ß-actin membrane and ß-actin cytosol lysates in *P2x6*^*-/-*^ and *P2x6*^*+/+*^ mice. Data (n = 3) represents mean ± SEM and are expressed as the % of total band intensity.

Since P2x6 has been implicated in regulating both Na^+^ and Mg^2+^ transport in the kidney. The renal expression of several sodium and magnesiotropic genes was analyzed in the two mice groups using RT-qPCR (Fig 3). Significant changes in the renal transcript levels of the apically expressed magnesium channel *Trpm6 were not detected*, neither where there expression differences for *Egf*, which regulates Trpm6 activity (Fig 3A and 3B) [24]. *Cldn16* was measured to investigate whether paracellular Mg^2+^ transport in the thick ascending limb of Henle’s loop (TAL) was affected in the P2x6^-/-^ mice. However no significant changes in *Cldn16* transcript levels were detected (Fig 3C). Since the epithelial Na^+^ channel (ENaC) activity is inhibited by purinergic signaling [9], its transcript levels were measured. *Scnn1* expression, encoding ENaC was the same in *P2x6*^*+/+*^ and *P2x6*^*-/-*^ litter mates (Fig 3E and 3F). Other Na^+^ and Mg^2+^ related genes expressed in the DCT cell such as *Slc12a3*, *Cnnm2*, *Kcnj10*, *Slc41a1*, *Slc41a3* and *Fxyd2* were also analyzed (Fig 4), since P2x6 was localized to this nephron segment. Again, there was no marked difference in expression levels between the two mice groups. To investigate whether other P2x subunits would compensate for the inactivation of P2x6, the genes encoding *P2x1-5* and *7* were also analyzed for changes in expression, but no quantifiable changes could be found (Fig 5). To ensure that the putative binding partners of P2x6, namely P2x2 and P2x4, did not compensate for the loss of *P2x6* at the protein level, Western blots for P2x2 and P2x4 were performed using membrane and cytosol fractions of P2x6^+/+^ and P2x6^-/-^ mouse kidneys. P2x4 was detected in both the membrane and cytosol, whereas P2x2 was only detected in the membrane fraction. Both proteins displayed a band at the expected height of ~54 kD (Fig 6A). After quantification no significant differences were found between P2x6^+/+^ and P2x6^-/-^ mice (Fig 6B).

### Increased P2x2 expression in *P2x6*^*-/-*^ heart

**Fig 7:**
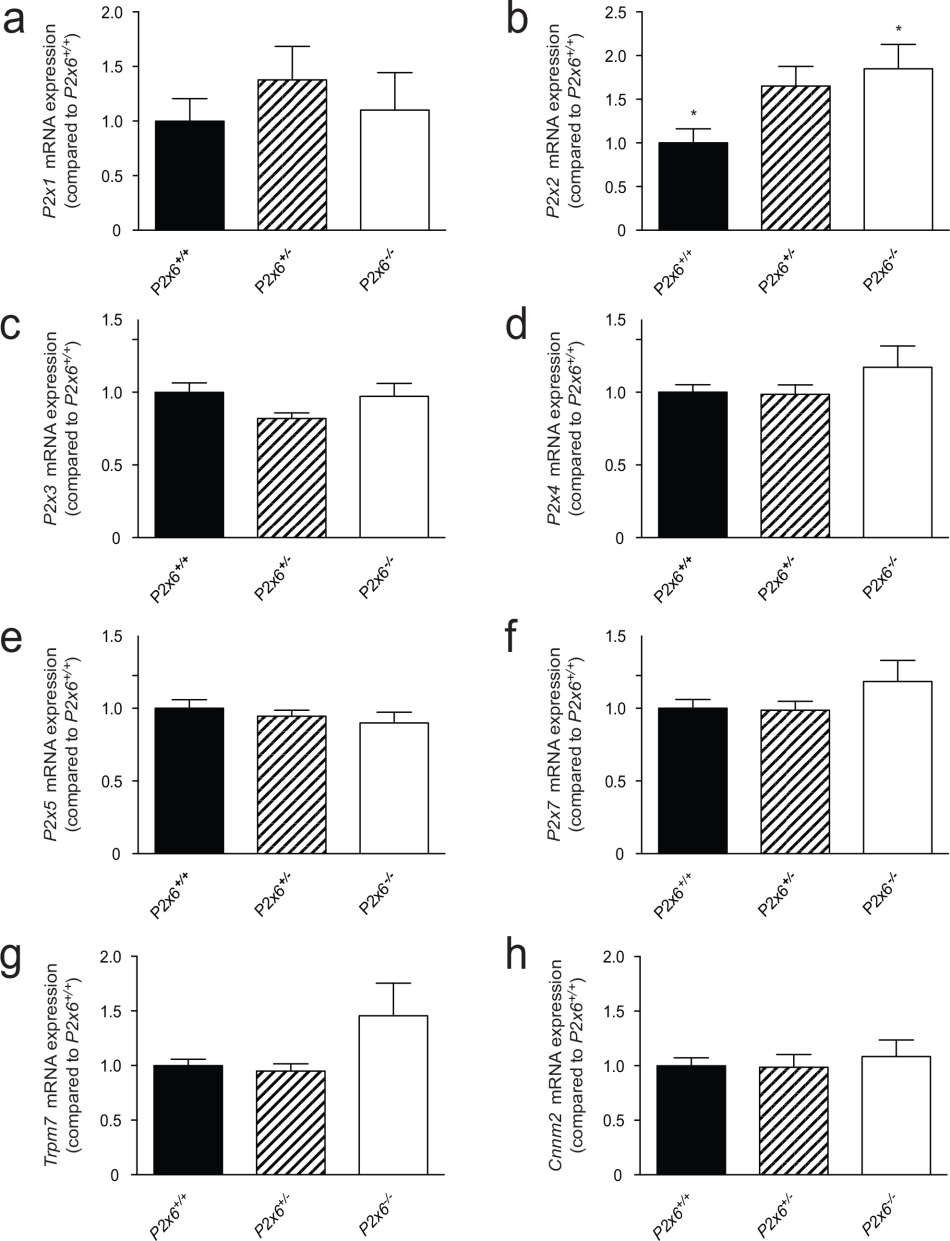
Compensatory mechanisms for the loss of P2x6 function in the heart. a-h) The mRNA expression levels of *P2x1* (a), *P2x2* (b), *P2x3* (c), *P2x4* (d), *P2x5* (e), *P2x7* (f), *Trpm7* (g), *Cnnm2* (h), in heart of *P2x6*^*+/+*^ (Black bars), *P2x6*^*+/-*^ (Striped bars), *P2x6*^*-/-*^ (white bars) mice were measured by quantitative RT-qPCR and normalized for *Gapdh* expression. Data represent mean (n = 10) ± SEM and are expressed as the fold difference when compared to the expression in *P2x6*^*+/+*^ mice. * P< 0.05 indicates a significant difference from *P2x6*^*+/+*^ mice.

Since upregulation of P2X6 has been linked to chronic heart disease [25], RT-qPCR analysis was performed on heart tissue of *P2x6*^*-/-*^ mice. As in the kidney, expression of the magnesiotropic genes *Trpm7* and *Cnnm2* remained unchanged. The *P2x2* subunit was however significantly upregulated two-fold (P<0.05) in the *P2x6*^*-/-*^ heart material compared to *P2x6*^*+/+*^ mice. *P2x* subunits 1, 3–5 & 7 did not show significant differences in gene expression (Fig 7).

## Discussion

This study demonstrated that P2X6 knockout mice have a normal phenotype, suggesting that P2X6 does not play a significant role in renal electrolyte handling. *P2x6*^*-/-*^ mice were physiologically similar to their wild-type littermates in terms of behavior, weight, food or water intake. No significant changes in serum concentrations or urinary excretion were observed for Na^+^, K^+^, Ca^2+^ and Mg^2+^ in *P2x6* null mice. Furthermore, loss of P2X6 function did not induce compensatory gene expression of any P2X purinergic receptors or other ion transporters that were investigated in the kidney. Likewise, no changes in P2x2 and P2x4 protein levels were detected in the kidneys of the P2x6 null mice compared to wild type littermates. Together, these results suggest that P2x6 is not involved in renal electrolyte handling under normal physiological conditions.

P2X receptors have been established as important negative regulators of ion transport in the kidney [26]. Specifically, homomers of P2X4 receptors and heteromeric complexes of P2X4 and P2X6 have been implicated in the reabsorption of Na^+^ and Mg^2+^ [9, 27]. In microdissected tubules of the TAL, extracellular ATP inhibited Na^+^ currents in a P2X-dependent manner [10]. P2X4 receptors also result in diminished TRPM6-mediated currents when overexpressed in HEK293 cells [18]. Moreover, several studies showed that P2X4 homomers and P2X4/6 heteromers inhibit ENaC in the CD, decreasing Na^+^ reabsorption [17] [27]. Although this vast amount of *in vitro* studies shows the importance of ATP in the regulation of renal ion channel activity, the physiological consequences remain undecided. In this study, we revealed that loss of P2X6 does not impair electrolyte handling *in vivo*, since renal electrolyte handling is unaffected in *P2x6*^*-/-*^ mice. Moreover, previous studies demonstrated that *P2x4*^*-/-*^ mice also displayed normal renal Mg^2+^ excretion [18]. These findings question the *in vivo* role of P2X receptors for renal electrolyte handling.

There are several explanations for the absence of a renal phenotype in *P2x6*^*-/-*^. First, the loss of P2X6 function may be compensated for by other P2x subunits. P2X6 functions in complexes with P2X2 or P2X4 and their function may counterbalance the loss of P2X6. However, our expression profile did not show differences in *P2x2* and *P2x4* expression at the transcriptional and protein level in *P2x6*^*-/-*^ mice. Second, *P2x6*^*-/-*^ mice might need to be challenged to reveal disturbances in Na^+^ or Mg^2+^ handling. For instance, in rat cardiomyocytes, *P2x6* expression is low under normal physiological condition [28, 29]. However, studies in patients have shown that P2X6 expression significantly increases after an ischemic cardiac event [25]. This latter study demonstrated that when the system is pathologically challenged, P2X6 expression can be induced. In our study, compensatory mechanisms, e.g. upregulation of other *P2x* subunits in the heart, were not detected in the *P2x6*^*-/-*^ mice, except for a significant increase in *P2x2* transcript. For now, which pathogenic factors would stimulate renal P2X6 expression are unknown. A transcriptome-wide screening, where mice were challenged with Mg^2+^-deficient diets, found no differences in *P2x4* and *P2x6* expression in the DCT [30]. Third, the mouse may not provide a good model to study the role of P2X purinoreceptors in the DCT. Although previous studies have shown that *P2x6* is the predominantly expressed *P2x* transcript in the DCT together with *P2x4*, P2X4 protein expression could not be confirmed in the DCT [18]. These findings together with our results shed doubt on the presence of functional P2X4/ P2X6 trimers in the DCT.

Over the last decade, the function of P2X6 has been widely debated [19, 31, 32]. Although recombinant homomeric P2X6 complexes have been reported to reach the plasma membrane in transiently transfected HEK293 cells [33], Others have shown that P2X6 requires the presence of P2X4 or P2X2 to become functional at the cell surface [31, 34–36]. This has been attributed to P2X6 lacking charged residues in the N-terminus, which prevents trafficking of the protein to the membrane [19]. Recent studies suggest that P2X6 may function as a nuclear regulator of post-transcriptional modifications in neurons [32]. However, nuclear localization has not been identified in other tissues [37]. Further *in vitro* studies are required to examine the function of P2X6.

In conclusion, *P2x6*^*-/-*^ mice exhibit an apparent normal physiological behavior and renal electrolyte handling under normal conditions. Serum Na^+^, Mg^2+^, K^+^ and Ca^2+^ concentrations were not significantly different compared to wild type littermates and compensatory expression of relevant electrolyte transporters was absent. Thus, P2X6 is likely not significantly involved in the regulation of renal electrolyte handling under normal physiological conditions. Our results, therefore, question the essential role of P2X6 in normal electrolyte homeostasis.

### Ethical Approval

All experiments were performed in compliance with the Central Animal Laboratory Nijmegen and the animal ethics board of the Radboud University Nijmegen (DEC #2013–185).

## Supporting Information

S1 ARRIVE Checklist The ARRIVE Guidelines Checklist Animal Research: Reporting In Vivo Experiments. (PDF)

S1 Table Primer Sequences for Expression Profiling. (DOCX)

S2 Table Primer Sequences for RT-PCR. Gapdh, glyceraldehyde 3-phosphate dehydrogenase; P2x1-5, 7, P2X purinoreceptors 1–5, 7; Trpm6, transient receptor potential cation channel, subfamily M, member 6; Cnnm2, cyclin and CBS domain divalent metal cation transport mediator 2; Egf, epidermal growth factor; Cldn16, claudin 16; Slc12a3, solute carrier family 12, member 3; Scnn1, Amiloride-sensitive sodium channel subunit; Trpm7, transient receptor potential cation channel, subfamily M, member 7; Fxyd2, FXYD domain containing ion transport regulator 2; Kcnj10, ATP-sensitive inward rectifier potassium channel 10; Slc41a1, solute carrier family 41, member 1; Slc41a3, solute carrier family 41, member 3.
(DOCX)
