# Supplementary material for: P2X6 Knockout Mice Exhibit Normal Electrolyte Homeostasis
Source: PLoS One. 2016 Jun 2;11(6):e0156803. doi: 10.1371/journal.pone.0156803 (PMC4890750; doi:10.1371/journal.pone.0156803)
Supplement: S1 Table — (DOCX) [file pone.0156803.s002.docx]

**S1 table.** Primer Sequences for Expression Profiling

| ***P2x6*** | **Forward** | **Reverse** |
| --- | --- | --- |
| **Exon 1** | 5’- AACAACACTGGCCTGAATCC -3’ | 5’- ACCCGACAGTTCCTGGTTAG -3’ |
| **Exon 1-2** | 5’- GTGCTAACCAGGAACTGTCG -3’ | 5’- CGGTTCTCCAGCTCCTTAAC -3’ |
| **Exon 1-3** | 5’- GTGCTAACCAGGAACTGTCG -3’ | 5’- CACGTTCTCTCCCTGTGATG -3’ |
| **Exon 2-6** | 5’- ACGTGGCTGACTTTGTGAAG -3’ | 5’- TGTGAAGTTCTTGGCCTGAG -3’ |
| **Exon 2-7** | 5’- AGCCATCACAGGGAGAGAAC -3’ | 5’- AGGCATTGGATCTGGAGAAG -3’ |
| **Exon 6-12** | 5’- TCAGGCCAAGAACTTCACAC -3’ | 5’- GTAGCAGCAGGTCACAGAGG -3’ |
| **Exon 8-12** | 5’- GACACGAAAGGCTCTGACTG-3’ | 5’- GGAGAGGGTGTAAGCCTCAG -3’ |
| **Exon 12** | 5’- CCTCTCCCTGCTGGTTAAAG -3’ | 5’- ACTTGGACCAGGATCGTCTC -3’ |
