# Supplementary material for: P2X6 Knockout Mice Exhibit Normal Electrolyte Homeostasis
Source: PLoS One. 2016 Jun 2;11(6):e0156803. doi: 10.1371/journal.pone.0156803 (PMC4890750; doi:10.1371/journal.pone.0156803)
Supplement: S2 Table — Gapdh, glyceraldehyde 3-phosphate dehydrogenase; P2x1-5, 7, P2X purinoreceptors 1–5, 7; Trpm6, transient receptor potential cation channel, subfamily M, member 6; Cnnm2, cyclin and CBS domain divalent metal cation transport mediator 2; Egf, epidermal growth factor; Cldn16, claudin 16; Slc12a3, solute carrier family 12, member 3; Scnn1, Amiloride-sensitive sodium channel subunit; Trpm7, transient receptor potential cation channel, subfamily M, member 7; Fxyd2, FXYD domain containing ion transport regulator 2; Kcnj10, ATP-sensitive inward rectifier potassium channel 10; Slc41a1, solute carrier family 41, member 1; Slc41a3, solute carrier family 41, member 3. (DOCX) [file pone.0156803.s003.docx]

**S2 table.** Primer Sequences for RT-PCR

|  | **Forward** | **Reverse** |
| --- | --- | --- |
| ***Gapdh*** | 5’-TAACATCAAATGGGGTGAGG-3’ | 5’-GGTTCACACCCATCACAAAC-3’ |
| ***P2x1*** | 5’-CCGAAGCCTTGCTGAGAA-3’ | 5’-GGTTTGCAGTGCCGTACAT-3’ |
| ***P2x2*** | 5’-CACCACCACTCGAACTCTCA-3’ | 5’-GGTACGCACCTTGTCGAACT-3’ |
| ***P2x3*** | 5’-GGTGGCTGCCTTCACTTC-3’ | 5’-TCAGCCCCTTTGAGGAAA-3’ |
| ***P2x4*** | 5’-TTGGCTCTGGCTTGGCGCTC-3’ | 5’-TCTCCGGAAAGACCCTGCTCG-3’ |
| ***P2x5*** | 5’-GAGCGAGTTTTACCGAGACAAG-3’ | 5’-GATGAACCCTCTCCAGTGGC-3’ |
| ***P2x7*** | 5’-GGGGGTTTACCCCTACTGTAA-3’ | 5’-GCTCGTCGACAAAGGACAC-3’ |
| ***Trpm6*** | 5’-AAAGCCATGCGAGTTATCAGC-3’ | 5’-CTTCACAATGAAAACCTGCCC-3’ |
| ***Cnnm2*** | 5’-GGAGGATACGAACGACGTG-3’ | 5’-TTGATGTTCTGCCCGTACAC-3’ |
| ***Egf*** | 5’-GAGTTGCCCTGACTCTACCG-3’ | 5’-CCACCATTGAGGCAGTATCC-3’ |
| ***Cldn16*** | 5’-GTTGCAGGGACCACATTAC-3’ | 5’-GAGGAGCGTTCGACGTAAAC-3’ |
| ***Slc12a3*** | 5’-CTTCGGCCACTGGCATTCTG-3’ | 5’-GATGGCAAGGTAGGAGATGG-3’ |
| ***Scnn1a*** | 5'-CATGCCTGGAGTCAACAATG-3' | 5'-CCATAAAAGCAGGCTCATCC-3' |
| ***Trpm7*** | 5'-GGTTCCTCCTGTGGTGCCTT-3' | 5'-CCCCATGTCGTCTCTGTCGT-3' |
| ***Fxyd2*** | 5'-TCAGCCTTTCTTGTGACTGG-3' | 5'-GGTCTTCCTGTGGCCTCTACT-3 ' |
| ***Kcjn10*** | 5'-CCGCGATTTATCAGAGC-3' | 5'-AGATCCTTGAGGTAGAGGA A-3' |
| ***Slc41a1*** | 5'-CATCCCACACGCCTTCCTGC-3' | 5'-CGGCTGGCCTGCACAGCCAC-3 ' |
| ***Slc41a3*** | 5'-TGAAGGGAAACCTGGAAATG-3' | 5'-GGTTGCTGCTGATGATTTTG-3' |

# Gapdh, glyceraldehyde 3-phosphate dehydrogenase; P2x1-5, 7, P2X purinoreceptors 1-5, 7; Trpm6, transient receptor potential cation channel, subfamily M, member 6; Cnnm2, cyclin and CBS domain divalent metal cation transport mediator 2; Egf, epidermal growth factor; Cldn16, claudin 16; Slc12a3, solute carrier family 12, member 3; Scnn1, Amiloride-sensitive sodium channel subunit; Trpm7, transient receptor potential cation channel, subfamily M, member 7; Fxyd2, FXYD domain containing ion transport regulator 2; Kcnj10, ATP-sensitive inward rectifier potassium channel 10; Slc41a1, solute carrier family 41, member 1; Slc41a3, solute carrier family 41, member 3.
